# Miscellaneous

**Published:** 1897-04

**Authors:** 


### MISCELLANEOUS.

### RECENT ADVANCES IN RECTAL SURGERY.

In *Mathews' Journal of Rectal and Gastro-Intestinal Diseases,* Dr. A. B. Cooke, of Nashville, reviews the late progress in rectal surgery; part of which follows:

Entering into the discussion of the subject in its more practical bearings, the questions of symptomatology and diagnosis naturally at once present themselves. Along these lines advances of the most far-reaching import have been made. We now recognize that every pain referable to the rectum and vicinity is not necessarily produced by "piles"; that constipation is not always due to "torpid liver"; that diarrhea in its most intractable form frequently springs from and is maintained by a rectal lesion, and that dysentery, especially the chronic form, is often falsely so-called. We have learned that the reflex nerve impulses emanating from the ano-rectal region are incomparably more powerful than those from any other source, and that they may and do manifest themselves in a great variety of complex phenomena. We have also discovered that these reflexes, though as a rule pathological in expression, admit of wide utility as therapeutic agents.

In a communication entitled "A New Method of Resuscitating the Still-born," which appeared in the *American Medico-Surgical Bulletin,* issue of February 1, 1895, it was my privilege to call the attention of the profession to an application the originality of which, so far as I know, has not been challenged. Dilatation of the sphincter ani was the method advocated. Many reports received since the appearance of the article have served to justify the claim made. Of course, like every other remedy, it has its limitations; but, employed in the case of a normal viable child, threatened with death from asphyxia, no instance of its failure has come to my knowledge.

Another noteworthy application of this principle is seen in the same simple procedure. In the whole broad range of medical practice no emergencies are encountered more alarming than the accidents of general anesthesia, and none which call for more prompt and intelligent action. A sufficient number of well-authenticated cases are on record to warrant the statement that in many of these conditions also the divulsion of the sphincter ani offers the speediest and most effective means of resuscitation. That person is dead indeed whose cardiac and respiratory centers will not respond at once and vigorously to the stimulation of a suddenly stretched sphincter.

The two illustrations cited suggest other conditions in which benefit might reasonably be expected to follow this maneuver. But during the past few years so many extravagant, not to say ridiculous, claims have been heralded in regard both to the pathology and therapeusis of rectal reflexes, that the entire subject has fallen into discredit. This is neither wise nor right. Conservatism in medicine is well enough, but prejudice is as bad as ignorance. In this day of strange measures and remarkable methods the only safe guide is to be found in the old scriptural admonition, "Prove all things; hold fast that which is good."

Constipation is, perhaps, the most universal and certainly, in most cases, the most unconquerable of all diseases. We have awakened to the fact that this is not the simple and harmless condition it was once regarded, and with a broadened knowledge as to its complex nature and wide-reaching effects have come to realize its great importance. We now understand that, while in some cases it is merely a symptom, it is usually entitled to be regarded a disease and treated as such. The cholagogue-purgative-enema method treatment has been to a large extent abandoned and measures substituted which are based on the more scientific idea of removing the cause. Among these measures may be mentioned regulation of diet, attention to the hygiene of defecation, and abdominal massage. In my remarks on the reflexes I intimated that rectal diseases sometimes have this indirect origin. This is notably true of constipation. In such cases the external sphincter is usually at fault. This is one of the most sensitive areas in the whole body, and a lesion so minute as to escape observation not infrequently gives rise to chronic spasm and irritability of the sphincter, which result in marked hypertrophy. These cases are quickly and often permanently relieved by thorough divulsion. At the same time a careful examination may be instituted and any pathological condition present receive appropriate treatment. The recognition of constipation as the primary cause of a large majority of rectal diseases marks a genuine and very important advance in rectal surgery.

In appearance anal fissure is the most insignificant of all lesions of those parts. As a pain producer, however, it stands correspondingly preëminent. The barbaric method of treatment instituted by Maisonneuve about the middle of the present century has been practically discarded. It consisted of introducing the hand into the bowel, closing the fists, and subsequently withdrawing it. As an effective procedure it was doubtless a success, but not infrequently must have been as disastrous as heroic. Now, when divulsion is deemed necessary, it is accomplished gradually and cautiously under the relaxing and humane influence of general anesthesia. But the practice originated by Boyer in 1818, in the majority of cases leaves nothing to be desired. This consists of placing the anus gently on the stretch with a bivalve speculum and incising the superficial fibers of the sphincter in the base of the fissure. A few drops of a four per cent. solution of cocaine injected just beneath the lesion renders the operation entirely painless.

The condition known as ulceration of the rectum has long been a reproach and a stumbling-block to the regular profession, and an unfailing source of revenue to the quack; the former, because of imperfect methods of examination and unsatisfactory results of treatment; the latter, because the credulity of a public ever eager to be humbugged, has allowed ignorance and unscrupulousness to be cloaked under this vague but well-sounding term. And in truth, among all the diseases to which the rectum is subject, there is none more difficult to treat. But we have learned that even the most refractory cases are curable if one unvarying rule is established and rigidly enforced: That rule is *rest*—absolute rest in the incumbent position. This point gained, the victory is half won. The parts being permanently relieved of the physiologic congestion incident to the erect posture, it only remains to apply carefully and intelligently the well-known principles upon which the treatment of similar lesions of the mucous membrane in other situations depends.

I have stated that along all lines rectal surgery has kept pace with the rapidly changing steps of modern progress. This is the rule. If an exception exists it is in reference to the treatment of internal hemorrhoids. Except as modified in method and improved in technique the treatment of this disease is the same to-day as in the time of Hippocrates. I refer to the method most generally employed, the ligature operation. The only method which may be considered a rival to this is the one devised by Smith, the clamp and cautery treatment.

In keeping with the prevalence and popularity of this complaint ("piles") many ingenious innovations in treatment have from time to time appeared. For one reason or another these are all open to serious objection and with singular unanimity the profession has frowned upon them. However meritorious, they are by no means established, and it would be inappropriate, as well as profitless, to accord to them special mention.

"The present treatment of rectal fistula has little that is novel to offer. Within narrow and well-marked limitations, the operation known as Lange's may be regarded an improvement. But it is only applicable to those cases which have a single straight sinus, and such a condition is seldom encountered. The questions of the relationship between fistula and tuberculosis is still *sub judice.* The tendency, however, is strongly toward advancement. The intimate mutual dependence of these conditions is not recognized in modern teaching except as a curious popular superstition. If, after careful examination it is found that anesthesia can be borne, no system of reasoning can establish the theory that two exhausting diseases are better for the patient than one. In these cases some modification of the usual treatment is doubtless advisable, but under the condition stated, the propriety of operation does not admit of question.

---

### NOTES ON THE TREATMENT OF FECAL FISTULÆ.

At the thirteenth annual meeting of the New York State Medical Association, which was recently held in New York city, Dr. Frederick Holmes Wiggin, of New York county, presented a paper with the above title. The chief cause of the occurrence of fecal fistula was stated to be the delay in resorting to operative measures to which patients suffering from typhlenteritis, or strangulated hernia were frequently subjected while their ailment was carefully diagnosticated. The view recently advanced by a writer on the subject under consideration, that the best treatment for this condition consisted in its prevention, was concurred in. But in the case in which this mishap had occurred, it was pointed out that if the opening was of small size, was located near or below the ileo-cecal valve, and no obstruction to the fecal current existed, operative measures might be deferred, as in most instances the opening would close in a short time spontaneously. On the other hand, if the bowel opening was of large size, was situated laterally, or some distance above the ileo-cecal valve, and was accompanied by the escape of a large proportion of the contents of the bowel, operative procedure for the closure of the opening should be speedily undertaken.

The histories of three cases successfully treated by surgical measures was cited. In two instances, the patients were inmates of the Hartford (Connecticut) Hospital, and were operated upon by Dr. Wiggin, by reason of an invitation which was extended to him by the medical board of that institution, after several previous unsuccessful efforts to close the bowel openings had been made. The occurrence of the fistulous opening was due in the first case to failure, and in the second case to delay in resorting to surgical treatment of typhlenteritis, from which disease both patients originally suffered. In the third case, the bowel opening was caused either by the pressure of the gauze used to drain the abscess cavity, or by an ulcerative process which originated from within the gut. In the first case, as the opening in the bowel was of large size, irregular in shape, and the gut was thickened and friable, the diseased portion of bowel containing the opening, about four inches in length, was excised, and the divided ends joined by the suture method of Maunsell. In the second and third cases, the bowel openings were situated in the head of the colon, and were in both instances closed by means of several rows of sutures, after which the omentum was drawn over the former site of the fistula, and retained in position by sutures.

In describing the technique employed, the writer laid much stress upon the following points, viz.: the thorough disinfection of the parts, including the interior of the bowel, with hydrozone; the closing of the intestinal opening, when possible, before the breaking up of the peritoneal adhesions and the opening of the general cavity; the removal of any existing obstruction to the fecal current; the disinfection of the bowel surface with a solution of hydrozone, before and after the placing of the sutures; the control of the oozing from the cicatricial tissue by the same means, and the closure by a single row of silkworm-gut sutures without drainage of the abdominal wound after the washing of the peritoneal cavity with saline solution, some of which is allowed to remain.

In concluding, the writer stated that ever since September, 1893, when he had proved the value of hydrogen dioxide as an effective antiseptic, which in proper solution did not unduly irritate the peritoneum, when followed by a six-tenths per cent. saline solution, he had little reason to fear the danger of causing septic peritonitis from the accidental escape of pus or fecal matter while operating; and that when this complication had occurred, it had been invariably successfully met by the use of hydrogen dioxide in the manner described in the paper. He advised the excision of the diseased portion of the gut in those instances where it had become much thickened and friable, and expressed the belief that with a clearer understanding of the objects to be obtained by operation—*i. e.*, the restoration of the integrity of the intestinal canal, as well as the closure of the opening in the bowel—future operations for the cure of fecal fistula would more frequently result successfully than they had in the past.

The paper was discussed at some length by Dr. H. O. Marcy, of Boston, and Dr. Joseph D. Bryant of New York county, who commended it, and, in the main, indorsed the writer's views.—Abstract from *Medical Record*, Oct. 24, 1896.

---

CERTAINLY medicine has its imperfections. There are not only diseases incurable in physic, but cases indissolvable in law, and vices incorrigible in divinity.—*Sir Thomas Browne.*

### THE CIVIL LIABILITY OF PHYSICIANS AND SURGEONS IN CASES OF MEDICAL MALPRACTICE.

"Malpractice or malpraxis may be defined to be bad or wrong practice—bad or wrong as to results, whether these be loss of limb by amputation, deformity, serious injury to health, or even nature." (*Reese, Medical Jurisprudence Toxicology.*)

We shall present in this paper a brief and condensed statement of some of the more important rules bearing upon the liability of physicians and surgeons to their patients in civil suits for the recovery of damages. With the other phase of the question, the criminal side, we shall not deal, on account of the lack of space. A consideration of the criminal responsibility of physicians and surgeons would involve a discussion of *feticide, infanticide and homicide.* While criminal suits against practitioners are not infrequent, they are not nearly so universal as civil suits for the recovery of damages.

The law recognizes three kinds of malpractice:

1. Wilful malpractice, which occurs when the physician knowingly administers medicines or performs an operation which he knows and expects will result in damage or death to the individual under his care—as in case of criminal abortion.

2. Negligent malpractice—including all those cases where there is no criminal or dishonest object, but gross negligence of that attention which the situation of the patient requires, as where a physician administers medicines while in a state of intoxication, from which injury would result to his patient.

3. Ignorant malpractice is administration of medicines calculated to do injury and which a well educated and scientific medical man would not use or administer.

A physician or surgeon should not contract to effect a cure. This always savors of quackery. However, if he so contracts, he must fulfill his contract or suffer the consequences. In case of a failure to cure, it will be no defense for him to allege that unforeseen contingencies occurred, as the law presumes him to have knowledge of such at the time of entering into the contract. Where he makes an express contract to cure he will not be allowed to show want of sufficient skill or dexterity. The law presumes that he had these qualifications when he undertook the case. Reese gives an illustration of this rule. He says: "If a physician undertakes by contract to cure a woman of abdominal tumor by the operation of ovariotomy, and death results through an unavoidable peritonitis, this will not excuse him." (Reese Juris. etc., 620.)

Usually there is no expressed contract between the physician and the patient. In such cases the physician or surgeon impliedly contracts to furnish reasonable care and skill and diligence.

While there is some difference of opinion as to what constitutes reasonable care, skill and diligence, there is a preponderance of authority to the effect that it is neither the highest nor the lowest degree of education or skill, but the average of these. In defining what constitutes such skill, Pennsylvania courts have said that it was such skill "as thoroughly educated surgeons ordinarily employed." Perhaps the best definition of this rule extant is the following: "The true measure is that ordinarily exercised in the profession by the members thereof as a body. That is, the average of the reasonable skill and diligence ordinarily exercised by the profession as a whole. Not that exercised by the *thoroughly educated*, nor yet that exercised by the moderately educated, nor merely of the *well educated*, but the average of the thorough, the well, and the moderate, all in education, skill, diligence, etc."

The degree of care and diligence required of medical men is not the same in all places. A country practitioner is not expected to possess and exercise the same degree of care and skill or diligence that a city practitioner should, who has access to hospitals, asylums and laboratories, but medical men are bound to possess and exercise an average degree of skill possessed by the profession generally in other localities.

Of course it will be understood that the courts by their holdings do not mean to intimate that country practitioners are inferior in ability, or even qualifications, to their city brethren; but from the nature of the case, more would naturally be expected of the city practitioner who has access to the more modern appliances and methods of medicine and surgery.

The law, in the absence of any statute to the contrary, does not favor any particular school of medicine, but physicians must practice according to the regulations of their particular school. If one is a regular, he must use the standard regular remedies; if he is a homeopath, he must use the prescribed homeopathic remedies. If a member of one school uses the remedies and treatments of other schools, and the patient is injured in consequence thereof, the physician will be liable for damages.

Plaintiff, in order to recover, must show that he was actually damaged. Proof that the defendant physician and surgeon did not use ordinary care, will not be sufficient. The rule in full is: "The defendant is not liable unless damages ensue. The implied liability of a surgeon retained to treat a case professionally, extends no further in the absence of a special agreement than that he will indemnify his patient against any injurious consequences resulting from his want of the proper degree of skill, care or diligence in the execution of his employment. The plaintiff in an action for malpractice, will recover only nominal damages if he shows no injury resulting from the surgeon's negligence or want of due care. —MAGRUDER, *Columbus Medical Journal.*

---

### THE STATUS OF THE ANTITOXIN.

Drs. H. M. Biggs and A. R. Guerard, of the New York Health Department, have made an elaborate report upon "The Use of Antitoxic Serum in the Treatment of Diphtheria under the Supervision of the New York Health Department," closing with the following conclusions:

We desire to bring out strongly and clearly the fact that it matters not from what point of view the subject is regarded, if the evidence now at command is properly weighed, but one conclusion is or can be reached, whether we consider the percentage of mortality from diphtheria and croup in cities as a whole, or in hospitals, or in private practice; or whether we take the absolute mortality for all the cities of Germany whose population is over 15,000, and all the cities of France whose population is over 20,000 (in France and Germany antitoxin has been more generally employed than elsewhere); or the absolute mortality for New York city, or for the great hospitals in France, Germany and Austria; or whether we consider only the most fatal cases of diphtheria—the laryngeal and operative cases; or whether we study the question with relation to the day of the disease on which treatment is commenced, or the age of the patient treated; it matters not how the subject is regarded or how it is turned for the purpose of comparison with previous results, the conclusion reached is always the same, namely, there has been an average reduction of mortality from the use of antitoxin in the treatment of diphtheria of not less than fifty per cent., and under the most favorable conditions a reduction to one-quarter, or even less, of the previous death-rate. This has occurred not in one city at one particular time, but in many cities, in different countries, at different seasons of the year, and always in conjunction with the introduction of antitoxic serum, and proportionate to the extent of its use.

Then, finally, there is to be added not only the experimental proof of the specific action of antitoxin in neutralizing the toxin of diphtheria, but also the overwhelming mass of evidence derived from the personal observation of the most distinguished practitioners of medicine of every country. There are to-day, in the whole civilized world, not more than three or four active opponents of the antitoxin treatment of diphtheria whose names were known to the medical profession before the introduction of antitoxin.

It may therefore be affirmed that the following facts have been demonstrated:

1. That diphtheria antitoxin, where generally employed, has reduced the mortality from diphtheria at least one-half.

2. That it has a distinctly favorable effect on the clinical course of the disease, shortening it and lessening its severity.

3. That the earlier the treatment is commenced the better the results obtained; the mortality, when adequate doses of antitoxin have been given within the first forty-eight hours of the disease, not exceeding five per centum.

4. That antitoxin is a specific against true diphtheria (*i. e.*, where the symptoms are due solely to the Klebs-Loeffler bacillus) and is less efficacious in mixed infections, but even in these forms of diphtheria is of decided benefit.

5. That it is not necessary to wait for a confirmatory bacteriological diagnosis, but that in every clinically suspicious case of membranous angina, especially in children, a medium dose of antitoxin should immediately be given, and repeated if required by further developments of the case.

6. That antitoxic serum is a remedy without serious after-effects in the doses which have ordinarily been employed (the after-effects, such as rashes, etc., being insignificant in comparison with the danger of the disease); that it has no injurious action on the kidneys, the heart, or the nervous system; that it does not entirely prevent albuminuria, heart-failure, and post-diphtheritic paralysis, because the effects of the diphtheritic toxin which has already entered the system before the administration of the remedy, no matter how soon the treatment is begun, are not always completely counteracted by the antitoxin, though there is every reason to believe that in sufficient doses it does prevent any further extension of the toxic action after its effects have been produced.

7. That the protection conferred by immunizing doses of antitoxin is almost absolute for a short period of time—*e. g.*, three or four weeks, when a sufficient number of antitoxin units is administered; and that with a high grade of preparation, where only small quantities of serum are required, the remedy is absolutely harmless even with the youngest infants.

8. That antitoxin, if not a specific cure for all forms of diphtheria occurring in the human subject, is by far the best remedy for the treatment of the disease.

To the critics of the antitoxin treatment we may repeat the words of a famous German poet:

> "The best critics in the world are they
> Who, along with that which they gainsay,
> Suggest another and a better way."

---

### COCAINE ANESTHESIA.

In *The Medical News* of December 12th, Dr. John A. Wyeth accounts for frequent unsatisfactory results obtained in the use of cocaine as an anesthetic, and describes a method of using it which seems to meet the requirements. He invariably uses a 4 per cent.

solution of cocaine, made in a saturated solution of boracic acid, for cutting through the skin. He introduces this in very minute quantities—a minim at a time—immediately under the epidermis into the sensitive or malphigian layer of the skin. This whitens the area with which it comes in contact, and provided it be in a part where the circulation is not readily controlled, he at once makes an incision with a sharp-pointed knife as far as the anesthesia has spread. The needle is then inserted one-quarter of an inch from this point, and another minim of the solution injected at the same level. Proceeding thus, an incision of the requisite length can be made through the skin and corium into the subcutaneous areolar tissue. On account of the lack of sensation in the subcutaneous areolar tissues, a weaker solution may now be used, and poisoning thereby be avoided, as absorption takes place freely in this tissue. The following are the Schleich solutions which the author recommends:

NO. 1, STRONG.

| | | |
|---|---|---|
| ℞ | Cocaine muriate................................................ | gr. iij. |
| | Morphine muriate.............................................. | gr. $\frac{2}{5}$. |
| | Soda chlorid...................................................... | gr. iij. |
| | Sterilized dist. water, or saturated solution of boracic acid, q. s.......................................... | ℥ $3\frac{2}{5}$. |

NO. 2, NORMAL.

| | | |
|---|---|---|
| ℞ | Cocaine muriate................................................ | gr. iss. |
| | Morphine muriate.............................................. | gr. $\frac{2}{5}$. |
| | Soda chlorid...................................................... | gr. iij. |
| | Sterilized dis. water, or saturated solution of boracic acid, q. s. to.......................................... | fl. ℥ 3. |

NO. 3, WEAK.

| | | |
|---|---|---|
| ℞ | Cocaine muriate................................................ | gr. $\frac{1}{6}$. |
| | Morphine muriate.............................................. | gr. $\frac{2}{5}$. |
| | Soda chlorid...................................................... | gr. iij. |
| | Sterilized dist. water, or saturated solution of boracic acid, q. s. to.......................................... | fl. ℥ $3\frac{2}{5}$. |

—*Medical Review of Reviews.*

---

### EARLY DIAGNOSIS OF CARCINOMA OF THE BREAST.

Dr. A. Marmaduke Shield, in a clinical lecture at St. George's Hospital, goes over this subject very carefully and clearly (*International Med. Mag.*, Cincinnati *Lanc.-Clinic*). The question involved in the accurate diagnosis of this condition is such an important one that a careful study of the systems, as observed in the large experience of Shield, is valuable.

He states that out of 750 cases of breast diseases of all kinds at St. George's Hospital, between the years 1875 and 1895, 353, or about one-half, were carcinomatous.

The examination should be made with the flat of the fingers, and not by roughly pinching up a portion of the gland. The following points are to be noted:

1. The age: the most likely age is between forty and fifty-five.
2. The character of the pain, which is neuralgic, not throbbing, and is not accompanied by heat or redness.
3. The onset is insidious.
4. The contour is rarely globular; usually it is obtuse or irregular.
5. The hardness is stony, and is one of the most striking and reliable characteristics of scirrhus.
6. The growth does not move in the breast substance, but moves with it.
7. Retraction of the nipple is an important symptom, but it may be absent.
8. The dimpling of and the pigskin-like condition of the skin are of the utmost value.
9. The presence of enlarged glands is important, but their absence is not proof that the disease is innocent.

In case of doubt the author is strongly in favor of exploratory incision. He believes that there are peculiarities of structure or of growth in certain individuals which may be sufficiently pronounced to become hereditary, and thus a predisposition to carcinoma may be found in some families. He calls attention to the fact that certain obscure conditions coming on in elderly women, such as an insidious pleurisy, hydro-thorax, supposed "rheumatic" pains about the thorax or bones, or, of still greater importance, spontaneous fracture, or severe pains in the spine, terminating in paraplegia, may be due to cancer, and that in all such cases the breast should be carefully examined, as the discovery of scirrhus nodule may explain a very mysterious illness.

He insists on a very careful examination of the breast, lungs, and abdomen, especially of the liver, for secondary growths, as having important bearings on the question of operation.—*Medical Standard.*

---

### SOUTHERN GEORGIA AS A WINTER RESORT.

Dr. Lucius W. Baker, while on a visit to Southern Georgia lately (March 9th) contributed the following letter to the Boston *Medical and Surgical Journal* (March 18th):

"THOMASVILLE, GA., March 9, 1897.

"MR. EDITOR:—While spending a few weeks among the pines of Southern Georgia I have been struck with the desirability of this portion of the country as a place of residence for those who find the Northern winter and early spring to be a time of special hardship.

"In the treatment of pulmonary and bronchial troubles it is, of course, admitted that suitable out-of-door life is of the utmost importance, and the locality selected should be one where the atmosphere is dry and sufficiently warm to enable the patient to spend nearly all of his time out-of-doors. It is not my intention to compare the various resorts for pulmonary invalids. I simply desire to call attention to a certain section which seems to me to be worth the notice of the medical profession.

"The city of Thomasville, where I am at present stopping, contains about 7,000 inhabitants, and is situated 260 miles south of Atlanta. It is 200 miles from the Atlantic and 53 miles from the Gulf of Mexico. The elevation is 330 feet, and it is surrounded by forests of long-leaf pine. The sea atmosphere can only reach it after traversing miles of pine woods, which sift it of its saline elements and lessen its moisture. The soil is a mixture of sand and clay, dries very quickly after a shower, and the natural drainage is excellent. There is almost an entire absence of swamp lands, which always load the air with dampness, and which is so noticeable in many parts of Florida. The average winter temperature is 54°; the average spring temperature is 67°; the relative humidity is 65 per cent.

"The dryness of the atmosphere and the soft, warm air, laden with the odors of the pine, have brought this place into considerable repute; and many Northern and Eastern people have erected fine residences and made this their winter home. Their presence changes the character of the place somewhat, and increases its desirability as a place of residence; while largely through their influence the city has been provided with artesian water-works, a system of drainage, and other necessary improvements.

"The summers are long, and the heat must be enervating; but from December to May the climate is delightful, and must be beneficial to those who need to escape from the rigors of a New England winter."

---

### VAGINAL LIGATION OF THE UTERINE ARTERIES FOR UTERINE FIBROMATA.

Dr. Austin H. Goelet, Professor of Gynecology in the New York School of Clinical Medicine, in a paper presented to the New York Obstetrical Society (*Am. Gyn. and Obs. Journal*), maintains that his operation, to be successful, should be done in carefully selected cases only, and that the uterine arteries should be divided instead of simply being ligated, to secure complete obliteration of the vessels which supply the tumor with nourishment. Simple ligation is not considered sufficient where the tissues of the broad ligament are included in the ligature. When the vessel is not isolated the ligature does not always rupture the internal coat which is essential for complete obliteration. Shrinkage of the included tissues occurs from compression of the ligature which loosens and the circulation through the vessel is often restored.

The operation should be limited to interstitial growths which do not extend above the level of the umbilicus, small sub-peritoneal growths which spring from the uterine wall below the fundus and where extensive adhesions with adjacent organs have not formed through which the tumor may receive nourishment.

In those of the author, where the arteries have been divided, the result has been most satisfactory; that is, hemorrhage has been permanently controlled, menstruation has become normal, the other symptoms have disappeared and the tumor has atrophied to such a degree that it could be detected by careful bimanual examination, the uterus reducing to normal size.

The chief advantages in favor of the operation in properly selected cases may be enumerated as follows, viz.:

1. It is devoid of risk, and the peritoneal cavity is not opened.
2. It is easily done.
3. It confines the patient to bed for two weeks only.
4. It removes all symptoms produced by the tumor.
5. It effects marked diminution in the size of the tumor, which, in some instances, at least, entirely disappears.
6. It does not in anyway interfere with a hysterectomy, should it subsequently become necessary.
7. It does not unsex the patient.
8. The result is manifest within six months and the patient is not disabled nor inconvenienced by the operation.

---

### LAPAROTOMY FOR PERFORATING TYPHOID ULCERS.

Dr. Joseph Price, of Philadelphia, in the *Medical and Surgical Reporter* for November 7, 1896, reports three instances of perforation due to typhoid fever in which he operated and in which recovery followed.

When perforations occur, the patients may already be in so low a condition, owing to the toxic influence of the disease itself, that any operative interference may seem to be nothing but a hastening of the inevitable end, and hence it may be considered that some degree of selective care should be exercised in deciding whether or not to operate in any given case. Yet we must remember what the probabilities are in case of non-interference. On the one hand there is nearly certain death, while on the other there is at least a bare chance of recovery. There is here no room for timidity; desperate chances must often be accepted, and if we are rewarded with a most scanty percentage of recoveries we must surely feel that some measure of success has been attained, and that lives have been saved that otherwise would have been lost. Hence, although in longer series of cases than that presented by Dr. Price we can never attain the splendid results that he has just published, we feel confident that intervention in intestinal perforation is not only justifiable but obligatory, and that those who abandon every such case to the utter hopelessness of non-interference fail in the accomplishment of their duty. As Dr. Price happily expresses it, "the difficulty lies in timidity and over-sensitiveness as to the professional repute." As a matter of fact, we can only base our decision upon the general aspect and condition of the patient, and upon the chances which our clinical experience in the observation of intra-abdominal lesions reveals to us. From an extensive quotation made by the author from Dr. J. C. Wilson we take the following: "I take it for granted that almost every case of free extravasation of intestinal contents, however small in amount, into the peritoneal cavity terminates fatally. There is little reason to believe that any case of this kind recovers." In the light of our knowledge of such matters we can hardly conceive of any opinion at variance with the one just quoted. The question before us practically resolves itself to whether we can reconcile it with our own ideas of professional duty to allow our patients to die without an attempt being made to save them, however remote the chances of recovery under operation may appear to be.

---

### MORTALITY AMONG NEGROES.

In *The Sanitarian* for December, 1896, Dr. J. C. LeHardy, Savannah, Ga., says it is generally understood that the high rate of mortality among negroes is due to peculiarities of the race, and is therefore unavoidable. When negroes were slaves their masters, as a rule, preserved strict sanitary precautions among them, it being to their interest to do so. But since their emancipation, on account of the struggle for existence, their dense ignorance, and the spread of syphilis among them due to their lack of restraint, and also to the apathy of officials, their condition from a sanitary standpoint has been of the poorest, and doubtless to this is largely due the high death-rate prevailing among them. In Savannah the rate has increased, according to the statistics of Dr. William Duncan, from 23.6 per 1000 in 1858 to 35.5 in 1868, while among the whites it has decreased from 35.5 to 18.3 per 1000.—*Medicine.*

### ARGONIN IN THE ACUTE STAGE OF GONORRHEA.

Swinburn (*Journal of Cutaneous and Genito-Urinary Diseases*, August, 1896,) records 51 cases of acute gonorrhea which he has treated with argonin. This drug is a combination of silver with casein; it is a white powder, which, when heated over a water-bath, forms an opalescent, viscid, albuminous fluid, the maximum strength of which is 10 per cent.

The routine treatment of these cases was as follows: If only the anterior urethra was involved, only so much of the canal was irrigated with a weak (1-6000) solution of potassium permanganate; the patient was then placed on a table and the anterior urethra was slowly filled to distension with the 10 per cent. solution of argonin, which the patient was made to retain by holding the lips of the meatus together for five or ten minutes.

When posterior urethritis was present, intravesical irrigation with the permanganate solution was practiced from the meatus; after which a soft rubber catheter, about 15 French, cut off so that it was only seven inches long, was lubricated with argonin, slid down past the "cut-off muscle," and two drachms of the argonin solution were injected; the catheter was then withdrawn and the anterior urethra was filled with argonin as in anterior urethritis. No internal medication was given except to regulate the bowels.

This treatment caused a rapid diminution of the discharge, and, in the majority of cases, a corresponding diminution in the number of gonococci present.

The injections, which were repeated daily, were painless and were followed by no inflammatory reaction; on the contrary, the inflammation of the disease, *per se*, was markedly diminished from the very start; even in the earliest stages the ardor urinæ was markedly lessened, and in most cases gave no further trouble.—*Codex Medicus.*

---

FOR chapped hands and face and sore nipples:

| | | |
|---|---|---|
| ℞ | Compound tincture of benzoin. .......................... | 10 mm. |
| | Alcohol...................................................... | ʒ ij. |
| | Aquæ rosæ.................................................. | 30 mm. |
| | Glycerin..................................................... | ʒ j. |

Mix. Apply to chapped surfaces at night, after washing with soap and water and carefully drying.

—*Medical and Surgical Reporter.*

